# Bibliographical Notices

**Published:** 1841-10-01

**Authors:** 


### Periscope;

OR,

### CIRCUMSPECTIVE REVIEW.

" Ore trahit quodcunque potest, atque addit acervo."

### Notices of some New Works.

A New Operation for the Cure of Amaurosis, Impaired Vision, and Shortsightedness. In an Letter, addressed to John Richard Farre, M.D. &c. Physician to the London Ophthalmic Hospital, Moorfields. By *James J. Adams,* F.L.S. G.S. Member of the College of Surgeons. 8vo. pp. 50. London: John Churchill.

The division of the recti muscles of the eye would seem to be still the rage. The gentleman before us has applied it to the treatment of *amaurosis.*

He refers the origin of many cases of this complaint to a peculiar affection of that portion of the optic nerve which intervenes between the optic foramen and the ocular globe. He cautions us, however, that the term *amaurosis* means with him simply a dim or darkened sight, without implying any organic disease of the optic nerve. " Having thus," he adds, " restricted the term amaurosis to the expression of a symptom, common to many diseases of the eye, namely, dim or darkened sight; a necessity arises for distinguishing, by an additional term, the different cases of impaired vision in accordance with their degree or cause; therefore, as the *cause* of the amaurosis, which will form the subject of the following pages, will be found to depend on muscular action, and not on any disease or change of structure in the nerve, I propose to distinguish it from all the other forms of blindness by the term *Muscular Amaurosis.*"

His account of the symptoms is as follows:—

" In the simple forms of the disease the *objective* symptoms are few, they may be subdivided into two, namely, those which exist within the eye, and those which relate to its position and motions. Of the former very little can be said, since they consist only of slight variations from the natural condition of the pupil; the iris may be more dilated and less active than natural, and the colour of the pupil may be somewhat less brilliant than usual, though not to that degree which is recognized as a turbidity of the humours. Of the latter more may be observed; the position of one eye, and generally of the one most affected, may be slightly diverged so as to constitute a peculiar stare, but not a squint; also if the eye least affected be closed, the affected one may become violently inverted, which will be increased by any effort on the part of the patient to straighten it by looking directly forwards, though he may have perfect power to direct the eye to the outer canthus if he wills it; again, if both eyes be open and an object be held directly in front of the root of the nose, one eye may be apparently attracted towards the object, or turned towards its inner canthus, while the other will be repelled or moved towards its external canthus, so that the patient may be found to have scarcely any power over the equal convergence of both eyes.

Though these symptoms are frequently found either separately or conjointly, it is not unfrequent to find them all entirely absent, and only the natural appearances of the eye present.

The *subjective* symptoms are, in the uncomplicated forms of the affection, numerous and liable to much variety, and may like the *objective*, for convenience sake, be subdivided into two kinds, namely, those which affect the sight, and others which affect the feelings of the patient. Those which affect sight may commence in several different ways, either suddenly, gradually, or irregularly, in one or both eyes at the same time. If suddenly in both eyes, the patient may, while following some ordinary occupation, or on his first rising in the morning, find himself blind or nearly so; if one eye only be affected, he will feel conscious of seeing objects on the affected side less perfectly than on the opposite; this condition may rapidly pass away, or continue for several hours, then gradually cease or lessen; but on the first active exertion or application of the eye to fine work, or reading, the patient is surprised to find that he cannot see small objects for more than a few minutes at a time before they appear misty and confused; it will sometimes not advance beyond its first degree, but remain stationary for many years; at others, it will advance very rapidly, and the sight become so defective that the patient cannot see to guide himself about, though the amaurosis will very rarely be found complete, except for during a few hours at a time. In some instances the first failure of the sight is attended by a bright yellow or red spot, or, indeed, a mixture of them both; the yellow being encircled by the red.

If the disease commence gradually in both eyes, the patient at first will merely complain of the eyes feeling weak and soon fatigued after moderate exertions; this state may continue without any particular attention being paid to it, till at last he finds that on waking in the morning the sight is very dim, and remains more or less so during the time of his dressing, and that in the day time it has become much worse than it used to be, distant objects are less distinct, and near objects cannot be viewed for so long a time without a dimness and confusion of vision being produced. If he attempts to read, the book is brought closer to the eyes than is usually natural in order that he may see the print distinctly, and this he may do for the space of ten or twenty minutes, when the sight will become gradually dim, the letters confused, and (as it were) running one into the other: if the patient continues to look at the print for the space of a few seconds, it will become distinct, then misty, again distinct, and again misty, till at last the mist will increase and remain to such an extent that the form of the largest letter cannot be seen, and he will become for a short time more or less blind. However, if the eyes be closed for a few seconds, the sight will be again restored, and the patient once more enabled to see to read distinctly; though instead of being able to read for twenty minutes, before the symptoms of confusion and dimness of vision commence, he will find that he cannot read during more than *ten* or *fifteen* before another rest will be required, and that of a longer duration than the first, even perhaps, to three or four minutes; at the end of which time the sight will again become fit for reading, but for a still shorter period: perhaps not more than five minutes will elapse before the confusion of the letters will require another period of rest, of a still longer duration than the preceding; thus the increasing inability to maintain a clear view of an object will continue, until at last the patient will find it impossible, even after a long rest, to see any near object for more than a few seconds together; he is therefore compelled to give up, for many hours, or for the remainder of the day, the employment of his eyes on the subject which causes the dimness. When the disease has advanced to this state, he, in all probability, cannot see to read a single line of print by candle light; should it have commenced gradually in one eye the inconvenience will be somewhat less than above described. The gradual form of the disease, like the sudden, is always liable to a rapid increase in its severity."

Some cases follow, and they are succeeded by an account of the operation performed by Mr. Adams:—

"The operation which I propose, for the cure of muscular amaurosis, consists in the division, and *the extensive separation* of one, two, or more of recti muscles: of which, be it observed, the separation must be *equal* and *even* in each instance. The mode by which I perform this operation I have made peculiarly my own, by adapting a particular set of instruments to it, and by requiring, for its perfect success, a very extensive separation of the muscle, not only from the sclerotica, but from the cellular tissue and conjunctiva which lies in front of it. A description of the instruments I employ, with a detailed account of the steps of the operation may be seen in the Provincial Medical and Surgical Journal, No. 22, Vol. I. p. 356, and the instruments may be obtained at Weiss's."

The reason for Mr. Adams' proceedings will be found in this etiological paragraph.

"I venture to believe that no cause will be found so likely in its consequences to produce the peculiar disease of which we have been considering, as the arrangement of the recti muscles with respect to the nerve, for whatever the cause may be, it must possess the power of *acting suddenly*, of varying its degree by becoming *complete or incomplete* by turns, and of *changing from eye to eye; moreover, of existing without danger to the life of the patient, and, frequently, without in the least degree altering the healthy and natural appearances of the eye, from childhood to old age.*

I am therefore of opinion that the peculiar form of amaurosis, here designated by me as *Muscular Amaurosis,* depends on the bending or partial folding, and compression of the optic nerve, caused by the shortening and thickening of the recti muscles during a state of morbid contraction, which further may be attributed to an affection of the third and sixth nerve, probably at or near to their origins."

Those who would know more about the matter we must beg to refer to the pamphlet itself.

---

The Oration delivered before the Medical Society of London, at their Sixty-Eighth Anniversary, March 8, 1841. By *W. D. Chowne,* M.D. Member of the Royal College of Physicians, London, &c. 8vo. pp. 71. London, 1841.

To those who know Dr. Chowne, a complimentary mention of him will appear unnecessary. Few members of the Medical Societies of London have distinguished themselves more honourably by zeal for the advancement of medical science. The oration before us is highly creditable to its author, and gave great satisfaction to the body that it was addressed to. We are tempted to notice one passage in particular, because it embodies our own sentiments, and reads, we think, an useful lesson of scepticism in these credulous days. Not that we deem credulity more rife than heretofore—there was no need of that—but it is almost *as* rife as ever, and much *too* rife for the 19th century. The following is the passage to which we alluded, and which we may offer as a sample of the whole.

"The pleasure which arises from the belief that a new remedy has been discovered, the charm of novel modes of treatment, the reliance which bold and confident assurances are calculated to command, all conspire to take the judgment by surprize, and to obtain even for error, a place in the society of doctrines that are true, and sound, and based on the testimony of time, of experience and of strict impartiality. This association, however, is happily of but short duration, yet, short as it may be, it is injurious to the interests of the public, and disparaging to the character of our profession. In the outset of life, the young and the sanguine of all professions are liable to be misled by that which is the natural result of immature experience, too much hope and too much confidence; but however amiable and generous these may be, or however much they adorn the character, and are beautiful qualities in their relation to private life, they are not suited to the advancement of a grave, important, and complex science, such as that in which we are engaged; on the contrary, few things tend more to that object, than the exercise of sound and guarded discrimination, in the yielding or withholding of confidence, where new doctrines are the subjects of consideration.

Those who can look back, through a series of years, upon the phantasies which have been boasted into ephemeral fame, by the dupe, by the enthusiast, and by the empiric in turn; who have seen these visions come and go, ushered into being with *pretensions* the most *ostentatious*, but exemplifying, in their rapid lapse into neglect, their utter worthlessness and nothingness, will hold themselves more than justified for the exercise of that which the world calls scepticism, and for increasing their reserve, in proportion as the claims upon their faith, may have been confidently and authoritatively advanced; they may perhaps, deem themselves excused, should they, with more than common freedom, entertain the opinion that suspicion ceases to be a failing, where the questions relate to a science of facts, and where the character of the science, and the public health, (of which that science is the accredited, and should be the faithful guardian,) are involved in the issue."

---

MEMORANDA ON FRANCE, ITALY, AND GERMANY, WITH REMARKS ON CLIMATES, MEDICAL PRACTICE, MINERAL WATERS, &c. To which is added an Appendix on some of the Predisposing Causes of Disease, and on the Advantages of Travel and a Residence Abroad. By *Edwin Lee*, Esq. M.R.C.S. &c. 12mo. pp. 342. London: Saunders and Otley, 1841.

MR. LEE is very favourably known by his writings on the Spas of Germany. He is an intelligent English Surgeon, observant of what presents itself, and judicious in reflecting on it. All we can be expected to do is to notice a few points, which may seem to possess utility or novelty.

*Climate of Paris.*—The principal advantages which Paris has over London consists in the greater purity, clearness, and dryness of the atmosphere, its freedom from smoke and fog, and in the weather being less variable from day to day, as the summers are hotter, and the winters equally cold, if not colder. The average quantity of rain throughout the year, Mr. Lee conceives to be as great in Paris as in London. It would not, therefore, be advisable to select Paris as a winter residence for persons labouring under pulmonary complaints, (except perhaps some cases of asthma and sympathetic bronchial affection,) or rheumatism. Paris agrees very well with many dyspeptic invalids, to whom the light cookery of the French *cuisine* is better suited than the more substantial fare usually met with in Britain, which requires greater powers of digestion, always provided the invalid abstains from ragouts, rich sauces, or highly-seasoned dishes, and from indigestible vegetables, as truffles or mushrooms. The valetudinarian who labours under depression of spirits, combined with disordered digestion, would likewise frequently find himself better after a few months sojourn in Paris, which offers more resources for mental relaxation and amusement than any other city, not excepting London. Baths are also more general, which is a great advantage to invalids.

Among the most prevalent diseases of Paris may be enumerated inflammations of the respiratory organs, (especially in the winter and spring,) consumption, typhoid fevers, intermittents, rheumatism, chronic inflammation of the stomach and bowels, various forms of dyspepsia, and scrofula. Apoplexy, paralysis, and nervous diseases in general, appear much less frequent than in England; where also indigestion is more common, especially among the commercial and trading part of the population, and those whose avocations are of a sedentary nature, as clerks and others who have much writing or head-work.

*French and English Surgery.*—"I consider that the English treatment of the majority of surgical cases is also much preferable to that of the French and continental surgeons in general; and I do not speak from slight observation, when I state my conviction that many cases become the subject of operations in France which would be cured in England. While allowing the French to have great merit as skilful operators, I have no hesitation in stating, that their attention is too exclusively directed to local applications, and the performance of operations, as the means of removing surgical diseases; whereas comparatively little is known of the general and constitutional treatment by medicines, in which the British surgeons so much excel, and which so frequently obviates the necessity of having recourse to the more mechanical part of the art. Even diseases of the eyes, which are so obviously influenced by general treatment, were a few years ago, and are still to a certain extent, treated in Paris almost entirely by local applications."

*Disgraceful State of the Hospitals at Marseilles.*—The hospital which contains the foundlings is especially badly ventilated; the children being placed two, and sometimes three together in the same cradle, swathed up so as to prevent movement of their limbs, and the curtains of their cots closely drawn. I could not refrain from expressing to the physician who accompanied me, my astonishment and indignation at this mode of treatment, which must annually cause the death of a considerable number. He was disposed to lay the blame upon the administration, who would not grant a sufficient number of cradles; but it must surely have been in his power to prevent the nurses from altogether excluding the air, by keeping the curtains closely drawn around the cradles, which were scarcely large enough to contain the infants. The worst managed of these establishments, however, is the hospital, or rather, as one of the directors of the administration of hospitals termed it, the place of detention for the insane. This gentleman expressed himself ashamed to conduct me through the establishment, and not without reason, as the state in which it is allowed to remain reflects discredit upon all who have to do with it, and upon the town. The patients walk about, or are huddled up in corners of the courtyards, (the women's department being separated by a wall from the men's) around which are small cells, with iron grating instead of windows, many of them dirty, and containing one or two beds, with no other furniture. Some were even without beds, the occupant lying in a corner upon straw; chains were lately occasionally made use of as a means of repression, but the strait-waistcoat is now usually employed. At meal-times each patient receives his portion of soup or bread, eating it in the court-yard, or in his cell. A few cells on the first floor are fitted up as rooms for patients whose friends pay for their maintenance, and who are separated from the rest, and are better fed. No medical treatment appears to be adopted, except depletion in cases of violent mania.

*Peculiar agrémens of French Watering Places.*—Notwithstanding its scenic beauties, and the efficacy of its waters, Bagnères would not be an attractive summer residence to most English visitors, unless fond of seclusion, as the French, when *aux eaux*, associate very little with those whom they have not previously known. There are no public *reunions;* the accommodations in most of the French baths are very inferior to those of the German; and the presence of vermin in the beds, &c., is not unfrequently complained of. Each family is served with dinners from a *traiteur's:* there are few *tables-d'hôte,* and those only at the larger baths, for the accommodation of travellers. With the exception of two or three places, living is not dear at the Pyrenean baths; persons, for instance, may be boarded and lodged during the season at Bagnères de Luchon or Bagnères de Bigorre for five or six francs a day. Both England and Germany are, however, deficient in hot sulphur springs; and in cases where they are indicated, there are none in Europe (Aix la Chapelle perhaps excepted) which are so efficacious as those of the western Pyrenees.

*Bagnères de Bigorre* lies near the foot of the *pic du Midi,* and is the largest and most frequented of the Pyrenean watering places; a great proportion of the visitors remaining more for pleasure than for health. The number of English is at times not inconsiderable from Pau or other places in the South of France, or Italy. Many persons likewise pass some time at Bagnères, after having taken a course of one or other of the springs in this part of the country. It stands seventeen hundred feet above the level of the sea; is encircled on all sides but the north by green hills and pine-covered mountains, and is consequently one of the coolest Summer residences in the Pyrenees. The resident population amounts to eight or ten thousand, and the resources for amusement are greater than at the other baths. Bagnères was a great place of resort in the time of the Romans, by whom it was termed *Vicus aquensis.* There are numerous well-shaded walks and roads in the immediate environs; those which are most frequented are the *Allées* Bourbon, the garden Theas, and the valley of Campan.

The bathing establishments are numerous; the principal one belonging to government, termed the *Thermes de Marie Therese,* is an elegant structure of white stone, the interior of the bathing cabinets being composed of different coloured marble. The baths are thirty-six in number, and are exceedingly convenient, each having a dressing-room attached to it; over the door is inscribed the name of the spring by which it is supplied. Those of *La Reine,* which is the hottest, and the *Dauphin,* are most used. There is likewise every requisite apparatus for douche and vapour baths. As the water is too hot to be used at its natural temperature, it collects into large reservoirs at the back of the establishment, where it is cooled.

Frascati's is another bathing establishment, and also a lodging-house, which contains the public rooms for balls, billiards, and the newspapers. A single person may board and lodge here for about six francs a day. Other lodging and bathing-houses are supplied by particular springs, as the *Pinac, Lannes, Petit Prieur, Santé,* &c.

All these springs are saline, and in their composition are not unlike those of Baden-Baden or Bath, though they contain much less saline substance. Their temperature ranges from 27° to 35° R.

*What a delightful place Barèges is.*—No spot would seem to be less calculated for the situation of a bath than Barèges, which consists of a single street on the acclivity of the mountain, with the impetuous *gave de Bastan* foaming beneath. For nine months in the year the place is deserted, being left in the keeping of about twenty men, who pass the winter there to prevent its being occupied by the wolves, which not unfrequently take up their abode in the houses. As it frequently happens that the winter occupants are completely shut out from any intercourse with the valley by the snow, they are obliged to lay in a stock of provisions and fuel for three or four months, and on the return of fine weather, assisted by others from below, begin to clear the road, and ascertain the damage that has been effected; as many of the houses are every year carried away by the torrent, or overwhelmed by the avalanches. At the time of my visit in May, numerous workmen were employed in digging away the snow beneath which several houses were buried, and in getting the place a little in order for the season, which begins about the middle of June, from which period till September it is crowded with invalids, who certainly would not resort thither for pleasure, or unless they had learned from experience the efficacy of its mineral springs. This, by the way, may not be quite so certain.

In the centre of the street is a small square, on one side of which are the baths, on the other a military hospital, supported by government, for wounded soldiers, the number of which amounts to about four hundred, that of the visitors being about eight hundred, or a thousand, almost all of them invalids, and many who are not able to walk, or hardly to get about on crutches. There is only one table-d'hôte, and each party is served separately from a traiteur's; nor is there any society, and, as may be supposed, the climate is bad enough, so that existence in the place for several successive weeks would be unbearable, were it not for the benefit anticipated and experienced from the springs. In the bathing establishment there are sixteen private baths; but most of the patients bathe in the piscinæ or public basins. Douches are a good deal used, the water falling from a height of five or six feet from reservoirs through tubes about as thick as a man's fore-arm. The piscina for the hospital patients—but in which several visitors likewise bathe —is an oblong vaulted chamber with stone walls; the water is about three feet deep, and flows into the bath through a wooden trough, beneath which those who require the douche hold the affected part. About sixteen persons can bathe at the same time. The springs *la Douche, la temperée,* and Polard, are the most used; the temperature of the former is 44°, that of the second 33° R., and about 30° in the piscinæ, which are badly ventilated, and filled with vapour, which induces copious perspiration. There is likewise a piscina for indigent patients. The average number of baths required by patients is from thirty to forty. Though the springs are almost exclusively used for bathing, there is one drinking spring. They are strongly sulphurous, and more energetic in their action than any others in the Pyrenees, but they do not emit so disagreeable a smell as others of the same class, which contain a large proportion of sulphuretted hydrogen, as in these the sulphur exists principally in the form of sulphuret of soda. The cases in which they are most efficacious are scrofulous diseases of joints and other enlargements, rheumatic and paralytic affections, old ulcers and wounds, especially when there are foreign substances, as bullets, pieces of clothing, &c. lodged in the body, (the expulsion of which not unfrequently takes place during the course,) and some eruptions on the skin.

*Cauterets* lies in the secluded valley of Lavedan, surrounded by scenery of the most romantic kind. It chiefly consists in a single street, which widens out into a square. The resident population amounts to about fifteen hundred, and about two thousand visitors could be accommodated in the season, during which period it is generally thronged, especially by the Parisians, with whom it is a favourite summer retreat. Apartments are, consequently, often exceedingly difficult to be obtained, and are mostly very dear. In the square is the Hôtel du Cercle, where there are public rooms for reunions, but there is little general association among the visitors, so that a stranger who does not possess resources within himself, would, notwithstanding the magnificence of the scenery, find Cauterets but a dull residence. There are two or three tables-d'hôte, but they are not well attended. Cauterets has not the advantage of shaded walks, (the only rows of trees being in an enclosure behind the town, which serves for a promenade,) and from its being surrounded by lofty-peaked mountains, the summits of which are covered with perpetual snow, the reflection of the sun's rays must be, at times, severely felt, notwithstanding its elevated position; and what must be a great inconvenience to invalids, the bathing establishments, with one exception, are out of the town, some of them being at a distance of more than two miles.

The temperature of the springs varies from 21° to 40° R. The composition of the water is very analogous to that of other springs in this part of the Pyrenees, containing principally sulphuret of sodium, carbonate, sulphate, and muriate of soda, with carbonic acid and azotic gases in small quantities. The Raillière is the spring mostly used for drinking; it has a high reputation in pulmonary and nervous affections, and in dyspeptic cases.

Besides these springs, however, there are others in a different direction, which are much more sulphurous than the southern group, and are chiefly used for baths. The transitions of temperature are at times very great and sudden.

"Though the composition of some of these Pyrenean springs be not materially different, yet they differ considerably in the important circumstances of temperature and locality, and certain of them have acquired a special reputation in particular complaints. Thus, Barèges is recommended, *par excellence,* in cases of old wounds, ulcers, or long-standing affection of the bones; Bagnères de Luchon, in gouty, rheumatic, and cutaneous diseases; St. Sauveur in nervous complaints, and some local affections to which women are subject; while Cauterets, from the number of its springs, and the difference of their temperature, can be adapted to most of the above-mentioned indications. The greater number of patients are perhaps those labouring under the various forms of indigestion, and pulmonary complaints, or a tendency to consumption. The number of those with rheumatic and cutaneous diseases is likewise considerable."

How well adapted such a situation must be for the consumptive, we leave our readers to determine.

*Climate of Nice.*—Mr. Lee quite agrees with Sir James Clarke in the opinion that Nice is not suited for the Winter residence of consumptive patients, or those in whom there exists much irritability of the air-passages. The air is too sharp and exciting, and the occasional cold winds are severely felt. Those persons, however, in whom there exists a predisposition to consumption, or even those in the earliest stage of the disease, will often derive considerable advantage from passing November, December, and January, at Nice, provided there be not much acceleration of pulse or cough. The climate is generally of great service in chronic bronchial disease, particularly the catarrhal affections of elderly people, attended with copious secretion of mucus, and in those forms of asthma where there is little tendency to inflammatory action. I have known some persons labouring under these complaints who have passed several successive Winters at Nice. Patients with chronic gout, rheumatism, and paralysis, (the latter when not from apoplectic attacks,) as well as those whose general health has become deranged by a residence in tropical or unhealthy climates, will in general derive benefit from wintering at Nice; as will also many nervous hypochondriacal, and scrofulous patients, and those of a cachectic habit of body, with a languid circulation. Several of these cases will be likely to derive much more advantage from climate by the previous employment of mineral waters; the combination of these means offering, in my opinion, the greatest probability of cure and amelioration in long standing disordered states of the health, where a generally alterative and renovating treatment is indicated. I have, during the last seven or eight years, been in the habit of recommending many invalids, especially those with pulmonary complaints, to leave Nice before the middle of February, about which period inflammation of the lungs and bronchia is very common among the inhabitants. Several persons, by remaining throughout the Spring, have lost the advantage which they had gained during the preceding months; and though it may not seem to be a pleasant thing to travel at this season, yet, but little difficulty is experienced in proceeding to Rome or Pisa, as the coast-road to Genoa is always passable, and, except immediately after heavy rains, is in good condition. The constant passage of steam-boats likewise presents a rapid mode of conveyance to those who may prefer it.

Nice frequently disagrees with healthy persons of an irritable or plethoric habit, inducing headache or derangement of the digestive organs. The diet, both of those in health and invalids, wlll require particular attention, as several articles which agree very well in England, not unfrequently disagree with people at Nice. Wine, in particular, should be taken very sparingly. Those who remain during the Spring should avoid exposure to the sun's rays, by remaining in-doors in the middle of the day, or by carrying an umbrella, as there are no shady walks in the immediate neighbourhood of the town. Invalids should likewise avoid being out at sunset, as there generally arises an exhalation from the earth at that hour.

With respect to the choice of a residence, the Croix de Marbre appears to me to be the preferable situation for the majority of invalids. The villas in the neighbourhood of Cimiez are less convenient in many respects, especially with regard to dinners, which at Nice are usually sent from a *traiteur's*. When, however, a villa is preferred, one should be selected a little elevated above the plain, on account of the moisture which frequently arises from the irrigated meadows after sunset. The houses near the Corso, which have a southern aspect, are not objectionable, but those in the Piazza Carlo Alberto, and along the left bank of the Paglione, are less eligible on account of their northern aspect, and greater exposure to the winds, which sometimes sweep down the bed of the river with great violence. The *maison Gilli* is one of the best situated lodging-houses in the Croix de Marbre. There are also several new houses near the bridge not in an exposed situation. The *pension Anglaise* will be found a comfortable residence for small parties and single people.

*Hospitals of Florence.*—The hospitals here, as in many other parts of the Continent, are superintended by Government, and patients are admitted on application, without any other recommendation than that of their requiring medical assistance. The Spedale Santa Maria Nuovo is a handsome building, containing eight hundred beds; the wards are clean, spacious, and well ventilated. The professional treatment of the sick is in general judicious, and somewhat similar to the practice followed in France. Among the town practitioners, the abstraction of small quantities of blood is very common, which has frequently the effect of debilitating the patients, without effectually checking the progress of inflammatory attacks. Aperients, tonics, and other substances which are considered irritating, are generally avoided.

The Spedale di Bonifazio contains about the same number of beds as the other hospital, and is divided into two parts: one for the insane, the other for persons afflicted with incurable diseases, and for military men.

The number of insane patients in this hospital is about three hundred. Small cells, having each a window with iron bars, and containing a bed, are placed on either side of passages about fifty feet long. Each patient has a cell to himself, but in the daytime they are allowed to walk about the passages and in the open courtyards. All are clothed alike in a white woollen dress. The greatest attention is paid to cleanliness throughout the establishment; patients on their first arrival are isolated, in order that the peculiarities of their malady may be observed by the physician. When confinement of the hands is necessary, a wooden case (*manchot*) is used, into which both hands are placed, and confined by means of a strap passing round the waist. Furious patients are sometimes placed for a short time in a small darkened room well padded round the walls, the darkness and solitude being found to render them more tractable: moral measures are but little adopted in the treatment, though some of the patients are employed in mechanical occupations or gardening; the women in knitting, spinning, &c. The system formerly in use, of employing depleting measures every Summer, is now discontinued.

*Medical Practice of Rome.*—The treatment of disease inclines somewhat to that, which was prevalent in France some years ago; small bleedings are considered by many of the local practitioners as an almost universal panacea. In fact, a great proportion of the surgeons are little else than mere phlebotomists, and are but little calculated to treat either medical or surgical diseases. The physician is usually called in to prescribe for the constitutional disorder induced by surgical disease or by accidents; the part of the surgeon being limited to local applications or bandages. Tonics or exciting remedies (among which are reckoned aperients) are in general avoided, though in the malaria fevers large quantities of bark are given with advantage: sedative remedies, as prussic acid, henbane, &c., are not unfrequently exhibited, but the treatment of acute disease is principally of the expectant kind.

*Medical Treatment in the Neapolitan Hospitals.*—No particular exclusive theory is followed in the treatment of disease, different practitioners having different methods; but small bleedings, frequently repeated, are very general, as in other parts of Italy; and numerous sign-boards may be seen in the streets, at the barber-surgeons, representing the figure of a man with blood flowing in a full stream from the arms, legs, and neck. Stimulating, tonic, and laxative medicines are not frequently exhibited, but sedatives are more commonly used.

The most prevalent diseases are inflammation of the lungs and air-passages, consumption, rheumatic, gastric, and nervous fevers, and diseases of the eyes.

*Death of Homœopathy in its Native Land.*—"At the time of my former visit I was anxious to see the homœopathic hospital, of which I had previously heard, Leipsic being the head-quarters of this doctrine. I expected to have found at least forty or fifty beds filled with patients; but was rather surprised to find that the building (which is a small house in the suburbs) only contained eight, and even of these all but two or three were unoccupied. At my last visit to Leipsic, I understood that matters were going on badly with homœopathy, which indeed is now comparatively little heard of in Germany and France, and only required to be understood by the public for its absurdity to be apparent, though there will always be credulous individuals who are to be caught by any novelty, when presented under a specious appearance, and backed by an unintelligible name. During its whole progress it never was sanctioned by any individual of eminence in the profession, and was principally taken up as a means of acquiring wealth or a livelihood by persons who had never been previously heard of, or who were known as having failed to acquire practice by the honourable exercise of their profession; by whom every means were taken to puff it into notice, and to keep public attention directed to it; such as repeated histories of cures, the establishment of dispensaries, of which, I believe, the only one that remains is the above-mentioned at Leipsic, even if it be still in existence, for a few months before my arrival the house-physician having become convinced, during a residence of some time in the dispensary, of the nullity and danger of homœopathy, gave up his appointment, and published an exposition of the system pursued, with an account of cases, which clearly shows—what had long been evident to the bulk of the profession and the public,—that the so-called cures were recoveries from ordinary ailments by the efforts of nature, which were frequently a long time under treatment, whereas, by a proper medication and attention at the outset, they might probably have been removed in a few days, and that many of the more serious cases got worse instead of better, for the want of active treatment. It must not be supposed that the homœopathists always adhere to the principles of the doctrine. It has not unfrequently happened that persons who attributed their recovery to homœopathy were treated allopathically without their being aware of it. In fact, one practitioner in Leipsic, a professed homœopathist, candidly acknowledged that he pursued both plans of treatment, and was accustomed to ask his patients by which method they would be treated, as both were equally good."

We suspect that all homœopathists are not equally candid. The clever rogues prescribe allopathy, while they talk homœopathy. But the reign of any particular humbug (there is really no name so appropriate, albeit coarse) is short-lived—though the stock is so extensive that it is never worn out, and the market good enough to make it worth while to keep *some* article always upon sale.

We think our readers will agree with us that Mr. Lee's book contains a good deal both of amusement and of information.

---

### An Inquiry concerning the Diseases and Functions of the Brain, the Spinal Cord, and the Nerves. By *Amariah Brigham*, M.D. New-York. 1840.

The object of this useful little book is to direct attention to the diseases of the nervous system, diseases which have increased, and probably will still increase, with the advance of civilization.

The nature of the work precludes any thing like an analytical account of it. We may pick out, however, some passages for notice.

### *Division of the Skull into Regions.*

Dr. Brigham regrets that this has not been agreed on, for the purpose of rendering descriptions of injuries exact and clear. He recommends the following division of the cranium.

Let a line from the root of the nose to the middle of the back of the neck, corresponding to the longitudinal sinus, be called the *median line*. Let another from the meatus externus of one side be extended over the head to the other, called the *transvere line*. Then describe the injuries of the skull in reference to these lines, by inches and parts of inches.

For example, the precise situation of a wound would be made known, if said to be on the right or left side, and an inch and a half anterior to the transvere line, and an inch from the median. Its situation might, in some cases, be better understood by referring to other marked points on the head, as the eyebrows, and their angles.

### *Summary of the Results of Pathological Investigations.*

Dr. Brigham observes that the better part of what we know of the functions of the brain has been derived from pathology. Experiment has been peculiarly barren of results. The following is his resumé of what has been determined. We fancy that *it* must be taken with a reservation.

*First.*—That the cerebral lobes, or the hemispheres of the cerebrum, are the seat of intelligence.

*Second.*—That the cincritious portion of these lobes, probably, is, the seat of the mental faculties.

*Third.*—That the fibrous or medullary portions of the brain are connected with the motive powers, and transmit volition and sensation.

*Fourth.*—That the lobes of the cerebellum are not connected with the manifestations of the mental powers, but are with the motive; and appear also to be with the sexual propensity, and that the sympathy between them and the stomach is intimate.

*Fifth.*—That all the faculties of the mind may be manifested by one hemisphere of the brain.

*Sixth.*—That the different parts of the brain have different functions, and that the anterior portion of the cerebral lobes play the most important part in manifesting the mental powers, and appear to be the seat of the memory of words, events, and numbers.

*Seventh.*—That the striated bodies and the thalami are intimately associated with the motive powers of the extremities.

*Eighth.*—That parts in the middle and at the base of the brain, such as the fornix, corpus callosum, septum lucidum, pituitary body, and pineal gland, are not connected with the mental faculties.

*Increase of Inflammatory Diseases of the Brain.*

Dr. Brigham refers, as a striking proof of this, to the *Report of the City Inspector of the Interments in the City and County of New York* for above thirty years, from the commencement of making returns of deaths to the City Inspector in 1804 to the year 1838.

From the tables of the deaths from different diseases it appears that, while the population of New York has only quadrupled in the last thirty years, the deaths from inflammatory affections of the head, or from inflammation and dropsy of the brain alone, has increased more than twelve fold.

In the six first years of this period the deaths from these two diseases, which may be regarded as identical, were as follows:—

| | 1805. | 1806. | 1807. | 1808. | 1809. | 1810. |
|---|---|---|---|---|---|---|
| Inflam. of brain, | 17 | 16 | 15 | 17 | 18 | 12 |
| Dropsy do. | 16 | 22 | 30 | 28 | 28 | 42 |

| | 1833. | 1834. | 1835. | 1836. | 1837. | 1838. |
|---|---|---|---|---|---|---|
| Inflam. of brain, | 101 | 120 | 150 | 159 | 161 | 155 |
| Dropsy do. | 305 | 347 | 382 | 268 | 365 | 368 |

The population of the City of New York has risen during this time as follows:—

| | |
|---|---|
| 1805 | 75.770 |
| 1810 | 96.373 |
| 1815 | 100.619 |
| 1820 | 123.706 |
| 1825 | 166.086 |
| 1830 | 197.112 |
| 1835 | 270.089 |

It will be perceived that the most alarming increase has been of dropsy of the head, an inflammatory affection of the membranes of the brain and mostly confined to children.

It also appears that the same diseases have of late greatly increased in England and France.

According to the late Dr. Davis, of London, eight out of forty-five deaths, in the *Universal Dispensary* were produced by Dropsy of the brain, and Dr. Allison states that forty out of a hundred and twenty patients die of this disease in the *New Town Dispensary,* London. Marshall, in his Mortality of the Metropolis, London, from 1629 to 1831 says that since 1790 deaths from dropsy of the brain have been on the increase and to an alarming extent, the number being for the last few years about 800 per annum.

Dr. Coindet says that 20,000 deaths annually occur from the same disease in France.

Dr. Brigham very naturally attributes this to overdone cultivation of the intellect, on the part of the parent and offspring.

*History of St. Vitus.*

Perhaps this may be new to most of our readers.

"He, St. Vitus, was a Sicilian youth who, together with Modestus and Crescentia, suffered martyrdom at the time of the persecution of the christians, under Diocletian, in the year 303. The legends respecting him are obscure, and he would certainly have been passed over without notice among the innumerable apocryphal martyrs of the first centuries, had not the transfer of his body to St. Denys, and thence, in the year 836, to Corvey, raised him to a higher rank. From this time forth, it may be supposed that many miracles were manifested at his new sepulchre, which were of essential service in confirming the Roman faith among the Germans, and St. Vitus was ranked among the fourteen saintly helpers (Nothhelfer or Apotheker). His altars were multiplied, and the people had recourse to them in all kinds of distresses, and revered him as a powerful intercessor. As the worship of these saints was however at that time stripped of all historical connexions, which were purposely obliterated by the priesthood, a legend was invented at the beginning of the fifteenth century, or perhaps even so early as the fourteenth, that St. Vitus had, just before he bent his neck to the sword, prayed to God that he might protect from the dancing mania all those who should solemnize the day of his commemoration and fast upon its eve, and that thereupon a voice from heaven was heard, saying, ‘ Vitus, thy prayer is accepted.’ Thus St. Vitus became the patron saint of those afflicted with the dancing plague, as St. Martin, of Tours, was at one time the succourer of persons in small-pox; St. Antonius of ‘ those suffering under the hellish fire,’ and as St. Margaret was the Juno Lucina of puerperal women.”

*Influence of Attention on Bodily Organs.*

Dr. B. devotes a few pages to this subject. A not uninteresting nor unimportant one. He assures us that he has seen tumours, ulcers, and eruptive diseases increased and perpetuated, solely by mental attention directed to the disordered part.

Diseases of the eyes are thus increased, also diseases of the urinary organs. Bowel affections, particularly diarrhœa, are aggravated and sometimes caused by mental attention. During the prevalence of the Asiatic cholera in America, when every person had heard of the premonitory symptoms, there were but few who did not have some of these symptoms, especially griping pains in the abdomen, flatulence, diarrhœa, &c.

In prisons, where the discipline was such as to prevent any communication of intelligence from without, the cholera was not heard of, and did not prevail among the prisoners.

“ So strict is the seclusion, says Mr. Crawford,* in the Eastern Penitentiary, Philadelphia, that I found, on conversing with the prisoners, that they were not aware of the existence of the cholera which had but a few months before prevailed in Philadelphia.

To their ignorance of the existence of the cholera may doubtless be ascribed, in a great measure, their preservation from this disease, not a single convict having been attacked by it during the whole period that it prevailed in the city of Philadelphia, although the hospital for the reception of patients was in the neighbourhood of the prison. The powerful effect of alarm on the bodily system was singularly illustrated at this period at the Massachusetts State Prison. The chaplain having taken occasion one Sunday, from the pulpit, to advert to the ravages of the cholera, most of the prisoners who composed his congregation were, on retiring to their cells, seized with a complaint which it was greatly feared would lead to, but which happily did not terminate in, malignant cholera.”

Some of the disorders of the air-passages, of the larynx, trachea and bronchia, are kept up by attention. Some coughs are thus aggravated.

---

* Report of William Crawford, Esq. on the Penitentiaries of the United States, addressed to his Majesty's principal Secretary of State for the home department. 1835, p. 11.

Affections of the fauces and throat are kept up by the same cause. Every one knows that attention to the act of swallowing increases the difficulty of performing it. If there is some soreness of the throat, this attention produces more disordered action, more soreness, and more difficulty in swallowing and speaking. Dr. B. conceives that this often perpetuates soreness of the fauces and difficulty of speaking, of which clergymen have of late years complained.

This latter affection we have generally found occasioned by relaxation of the mucous membrane of the palate and fauces. Actors are liable to the same thing, which is occasioned no doubt by over exertion of the parts.

"In some instances," says the Doctor, "I have known the attention of clergymen, when thus affected with soreness of the fauces, and apprehensive of a protracted disease, to be diverted from their complaints by strong mental anxiety, and then recovery rapidly ensued. I have also known severe affection of the throat of the kind alluded to, disregarded by clergymen after a few days of ordinary medication, and have known them to preach with the soreness still upon them, and yet recover rapidly.

As I have said there are some cases of soreness of the throat and loss of voice depending on other causes, that are truly deplorable, often protracted for a long time and not unfrequently incurable. These arise from some affection of the brain, or of the nerves distributed to the organs of the voice. Sometimes the affection of the brain or nerves is only functional, arising from some overwhelming mental emotion, such cases may continue for a considerable time and then suddenly recover. Sometimes they yield suddenly to strong mental emotion, as anger. Laughter cured in one instance.

But in other cases the affection of the brain or nerves, that causes the loss of voice, depends upon some alteration of structure, and may be incurable. The injury of the nerves of voice, which prevents them from duly performing their functions, causes soreness and inflammation of the parts to which they are distributed."

Dr. Brigham remarks, that not only will mental attention aggravate, but it will also relieve disease. Kant was able to forget, by the strength of *thought*, the pains of gout and other diseases. The mental effort, he says, required great effort of the will and caused the blood to rush to his head, but never failed to afford relief.

The influence of the mind—of mental emotion, in causing and curing disease are altogether too much disregarded by medical men. While grief, fear, remorse, are as depressing as any measures we ever resort to, hope and faith are more powerful tonics than bark and wine. Innumerable are the instances that might be adduced in proof of this. Let the following suffice. When the plague raged at Messina, in 1743, the second of July, the Tutelar Deity, (Holy Mary, mother of God,) was taken down and carried in procession through the city. The plague stopped immediately.

In the life of Lord Chief Justice Holt, says Armstrong, a curious anecdote is recorded. When a young man, Holt had a flow of animal spirits which could not well be restrained, and he happened on one occasion, with some companions, to stop at an inn in the country, where they contracted a debt of such amount that they were unable to defray it. In this dilemma they appealed to Holt to get them out of the scrape. Holt observed that the innkeeper's daughter looked remarkably ill, and was told by her father she had an ague. Hereupon he gathered several plants and mixed them together with a great deal of ceremony, afterwards wrapping them in a piece of parchment, upon which he had scrawled certain letters and marks. The ball thus prepared he hung about the young woman's neck, and the ague did not return. After this, the never-failing doctor offered to discharge the bill, but the gratitude of the landlord refused any such thing, and Holt and his companions departed. When he became Lord Chief Justice a woman was brought before him accused of being a witch. She was the last person tried in England for witchcraft. She made no other defence than that she was in possession of a certain ball which infallibly cured ague. The ball was handed up to the judge, who untied it, and found it to be the same identical ball which he had made in his youthful days for the purpose of curing the woman's ague and paying his own bill.

Dr. Brigham collects several cases illustrative of the effects of mental excitement and depression. The following is, we dare say, known to many of our readers.

The late Mr. Pott was called in consultation on a surgical case: it was suspected that the patient had stone in the bladder. Mr. Pott examined him, found a stone, and abruptly said, I congratulate you on having your complaint perfectly known, for you may be cured by an operation. He observed a remarkable change in the patient's countenance, and having left him, went home. His assistant called in the evening and found the man dead.

In conclusion we think that the reader of this unassuming little work will obtain a good deal of instruction.

---

The Cure of Strabismus by Surgical Operation. By *William Mackenzie*, M.D. &c. Being an Appendix to his First, Second, and Third Editions of his "Practical Treatise on the Diseases of the Eye." 8vo. pp. 30. London: Longman & Co. 1841.

Mr. Mackenzie's reputation commands attention for everything which falls from him connected with ophthalmic surgery. The present brochure on the operation for strabismus presents us with a very useful account of all that has been ascertained upon the subject. It is quite a manual on the matter.

We shall select some portions for notice.

*Mr. Mackenzie's Method of Operating.*—The opposite eye being covered with a compress and roller, an assistant standing behind the patient, with the finger of one hand raises the upper eyelid, and with that of the other depresses the lower.

The operator, standing before the patient, desires him to turn his eye, as much as he can, in the direction which puts on the stretch the muscle about to be divided. If the case is one of convergent strabismus, he desires him to look outwards to his temple; if it is one of divergent strabismus, he desires him to look inwards to his nose.

We shall suppose the case to be one of convergent strabismus. The reader will easily conceive that many of the observations which occur in these pages regarding the cure of convergent strabismus, may be applied to that of the divergent variety by substituting *abductor* for *adductor*.

The steps of the operation, then, are as follows:—

1. With the forceps, the operator lays hold of the conjunctiva transversely, midway between the edge of the cornea and the caruncula lacrymalis, and raises it in a horizontal fold.

2. With the scissors, he snips this fold through vertically, along with the subjacent cellular substance, and then enlarges the incision, thus begun, upwards and downwards, so that it extends to half an inch in length.

The incision should not be nearer the cornea than half-way between its edge and the caruncula, lest in attempting to pass the blunt hook under the tendon, the operator find it impossible to do so, from the close attachment of the tendon to the sclerotica; nor ought it to be farther from the cornea, else the operator will require to penetrate deep by the side of the eyeball to reach the muscle.

The conjunctiva is merely to be slit up to the extent specified; it is not to be dissected from the subconjunctival fascia, nor is any portion of it to be cut away. In this way the wound will heal more readily, and the eye be less apt to protrude after the operation.

The incision of the conjunctiva is generally made in a vertical direction. In operating for divergent strabismus, it appears to be Mr. Elliot's plan to open the conjunctiva horizontally. Perhaps the incision made in this direction will gape less than a vertical one, but more separation of the membrane from the subjacent fascia will be required, to bring the tendon into view. A frænum will also be apt to form between the cicatrice of the conjunctiva and the external canthus.

3. The patient again everting the eye as much as he can; and the parts, if obscured with blood, being sponged, the operator insinuates the point of the blunt hook under the lower edge of the tendon of the adductor, and slides it up between the tendon and the sclerotica, till its point appears above the upper edge of the tendon. If there is any difficulty in bringing out the point of the hook at the upper edge of the tendon, from its carrying the fascia before it, the operator snips this through with the scissors, and frees the point of the hook.

In this part of the operation, unless the incision be nearer than usual to the cornea, or the operator take the trouble of removing a portion of the fascia, it is rarely the case that the fibres of the tendon are distinctly perceived. They are obscured by the fascia, which is now generally injected with blood. The operator, therefore, introduces the point of the hook where he thinks the lower edge of the tendon should be, and pressing it close along the surface of the sclerotica, he takes up on the hook every thing that lies between the sclerotica and the surface exposed by the incision of the conjunctiva. The hook, entering the cavity of the capsule, where the cellular connexion of the tendon to the sclerotica is naturally very loose, is easily passed beneath the tendon. This part of the operation, therefore, gives little pain, unless the hook is not sufficiently bent, or the bent part too long, so that it must be brought out over the eyelids, and by putting the muscle on the stretch, drag severely on the eyeball.

It is seldom that the patient is unable to evert the eye sufficiently, to allow the first and second steps of the operation to be performed, with no farther assistance than what has now been mentioned; but it sometimes happens that he cannot continue the eversion, at least to the necessary degree, to permit of the third step. In this case, the operator lays hold with the sharp hook, of the tunica tendinea, or, in other words, of the tendon of the muscle, where it is exposed through the incision of the conjunctiva, and without passing it deeper than the surface of the sclerotica, he moves the eye into the everted position. This he effects with a very slight degree of traction. He then intrusts the sharp hook, thus fixed, to an assistant, and proceeds to pass the blunt hook.

If artificial eversion is called for at the commencement of the operation, which sometimes is the case, especially in children, the operator passes the sharp hook through the conjunctiva and into the tunica tendinea, about one-fifth of an inch from the inner edge of the cornea, and having drawn the eye into the position required, intrusts the sharp hook to an assistant, till the first, second, and third steps of the operation are completed. After the blunt hook is passed under the tendon, the sharp hook may be removed.

At whatever step of the operation the sharp hook is used, it must be fixed in the tunica tendinea. It is useless to fix it in the conjunctiva; as this membrane, when we endeavour to move the eye by traction on the hook, yields, and slides away from the subjacent textures. To penetrate through the sclerotica with the sharp hook, is unnecessary.

4. The operator now takes the blunt hook in his left hand, and carrying the handle of it towards the temple, with the scissors he immediately divides, in ordinary cases, the tendon of the muscle from below upwards, and nearer the caruncula than where it is over the hook.

In this manner of operating, the muscle will most frequently be divided just where the tendinous part meets the fleshy fibres.

If the distortion is slight, the handle is to be carried over the nose, and the tendon divided nearer the cornea than where it lies over the hook, and close to its insertion.

If the distortion is great, the operator, before proceeding to use the scissors, should separate a considerable portion of the internal surface of the muscle from the sclerotica. Dr. Ammon does this by pressing the blunt hook repeatedly towards the cornea, and back again towards the caruncula. Mr. Elliot, for the same purpose, introduces a second hook, and steadies the eye by means of the one already under the tendon. The tendon being then drawn into view, the muscle is to be divided. If hypertrophied, a part of it should be cut out, which is best accomplished by passing a ligature under it with a blunt needle, tying the ligature upon the muscle, dividing the latter nearer the caruncula than where it is within the ligature, and lastly, cutting off the ligature, with a portion of the muscle which it embraces.

If, in the third step of the operation, only a portion of the tendon, and not its whole breadth, appears to be upon the blunt hook, the division with the scissors should not be immediately proceeded with; but, with another and smaller blunt hook, the operator should take up the remaining breadth of the tendon, and having divided this portion with the scissors, proceed to divide the principal portion, which he has on the first hook. It must be remarked, however, that when the tendon is raised and drawn forwards on the concavity of the blunt hook, it sometimes assumes a round and contracted appearance.

If the operator has any doubt about his having divided the whole of the muscle, he should not proceed to ascertain the position of the eye, and much less incautiously announce to the patient that the operation is finished, until he has examined with the blunt hook, and snipped across any portion which may have escaped. The mark of the semicircular insertion of the muscle, with its minute tendinous fibres, adhering to the sclerotica, will show distinctly that the muscle has been divided.

Such, then, is the operation for convergent strabismus. That for the divergent variety is generally considered rather more difficult, owing to the greater narrowness of the space between the eyelids, and the insertion of the abductor being farther from the cornea than that of the adductor. As to this, a good deal will depend on the size and prominence of the eye.

Upon the same general plan, the levator or depressor is to be divided, in cases of distortion upwards or downwards.

*Unfavourable Effects of the Operation.*—Some of the unfavourable effects which are apt to arise from the operation are trivial, but others are important.

1. The white cicatrice of the conjunctiva, in the situation of the wound, is of no moment.

2. The eye which has had its adductor divided, presents a greater gap between the cornea and caruncula than natural, the lids appear more open, and the eye more prominent and convex at the nasal angle. This arises from the drawing of the eye forwards by the two oblique muscles, which are no longer perfectly antagonized. If the plan of dividing part of the levator or depressor, in addition to the division of the adductor, were followed, the eye would be particularly liable to project unnaturally.

If both eyes have been operated on, both are rendered more prominent than natural, but being equally so, the circumstance attracts less notice. When only one eye projects, and the projection is great, the physiognomy is very remarkably and disagreeably affected. This affords a reason for operating on the opposite eye, if it presents the slightest degree of strabismus. If we venture to operate on a straight eye, or on one but slightly distorted, for the purpose of equalizing the projection of the two, the tendon should be divided, close to its insertion, and with as little separation of its cellular connexions as possible, for fear of eversion.

3. Whether one eye only or both have been operated on, double vision is not an unfrequent effect. In this case, parallelism of the eyes has not been perfectly restored. If the adductor of one eye has been divided, and the eye is thereby everted in any degree, double vision occurs when the patient looks straight forward, or towards the other side. In general, this effect gradually subsides as the eye recovers the power of adduction, by the action of the inner fibres of the levator and depressor, and the re-adherence of the divided muscle to the sclerotica. The patient should be instructed to look forward at objects, and to avoid looking to a side, especially to the side which increases the double vision, by causing divergence; to the left, for instance, if the right adductor has been divided. Except where each eye, being shaded in its turn, remains straight, tying up one of them only prolongs the evil.

4. One of the most annoying consequences of the operation is extreme eversion of the eye, when the adductor has been divided. Too great a separation of the muscle, and dividing it too far from the cornea, are the causes of this effect; which is still more apt to occur, if, in addition to these causes, the motions of the eye had been previously free and the distortion slight, or if both eyes have been cut. Eversion may also be brought on, soon after the operation, by the patient's looking too much to the side.

Extreme eversion is attended by a disagreeable expression of countenance, giddiness, and such a degree of double vision as unfits the patient for pursuing any employment, and even for walking about, with both eyes open.

The moment the surgeon observes a tendency to eversion, he should caution the patient against turning his eyes much to either side, and especially against such lateral motion as produces divergence of the optic axes; and should recommend him to look always straight before him, and to exercise his eye frequently on near and small objects. If the opposite eye does not become distorted on being closed, it should be bandaged for four or five weeks. If the eversion continues after this, the abductor should be divided. If the eye is inverted after an operation for divergent strabismus, the adductor may require to be divided, should such exercise of the eyes as tends to diminish the convergence, viz. looking forward at distant objects, prove unsuccessful. The division of the antagonist muscle, in either case, will allow the eye to resume its position in the centre of the orbit, and the lateral motions will be performed by the re-adhering muscles, and by the levator and depressor.

The mutual divergence which generally exists in cases of eversion, occurring after division of the adductor, may be remedied by operating on *either* abductor. In a patient operated on by Mr. Charles W. G. Guthrie, after the adductor of the inverted eye was divided, it gradually became everted. The case was now one of mutual divergence. After some weeks, the abductor of the better eye alone was divided, which cured the eversion of the other eye, without any interference with its abductor. Mr. Guthrie's explanation or theory is not satisfactory. He mentions that from a train of reasoning he was led to select the better eye, as an operation on it alone would cure the eversion. This is not the case. The division of the other abductor would with equal certainty have removed the mutual divergence, though objectionable on account of the prominence it would have left from the division of two recti of the same eye.

Alternating eversion, on looking to either side, sometimes follows an operation for convergent strabismus. After division of the adductor of one eye, for example, it has happened that the patient, on looking at any object placed straight before the eyes, directed the axes of both correctly, so that no obliquity could be detected, and vision was single. If, however, without turning his head, he regarded any object placed a little to either side, the eye of that side was instantly everted to a very considerable extent.

